# Effect of Cycling on a Stationary Bike While Performing Assembly Tasks on Human Physiology and Performance Parameters

**DOI:** 10.3390/ijerph17051761

**Published:** 2020-03-08

**Authors:** Atef M. Ghaleb, Tamer M. Khalaf, Mohamed Z. Ramadan, Adham E. Ragab, Ahmed Badwelan

**Affiliations:** 1 Department of Industrial Engineering, College of Engineering, King Saud University, Riyadh 11421, Saudi Arabia; tamkhalaf@ksu.edu.sa (T.M.K.); mramadan1@ksu.edu.sa (M.Z.R.); aragab@ksu.edu.sa (A.E.R.); A.BADWELAN@gmail.com (A.B.); 2 Department of Mechanical Engineering, College of Engineering, Al-Azhar University, Cairo 11371, Egypt

**Keywords:** EEG, ECG, human performance, neuroergonomics, seated work

## Abstract

*Objective*: This study evaluated participants’ ability to assemble a computer keyboard while at a cycling workstation. Depending on task completion time, error percentage, and workload based on subjective workload ratings, subjective body discomfort, electroencephalography (EEG) and electrocardiographic (ECG) signals, human performances were compared at four different cycling conditions: no cycling, low level cycling (15 km/h), preferred level cycling, and high level cycling (25 km/h). *Method:* The experiment consisted of 16 participants. Each participant performed the test four times (each cycling condition) on different days. *Results*: The repeated measure test showed that the alpha and beta EEG signals were high during session times (post) when compared with session times (pre). Moreover, the mean interbeat (R-R) interval decreased after the participants performed the assembly while pedaling, possibly due to the physical effort of cycling. *Conclusions*: Pedaling had no significant effect on body discomfort ratings, task errors, or completion time.

## 1. Introduction

Regular exercise contributes to managing depression, improving mental health, and preventing chronic diseases and premature death [1,2]. Additionally, it produces cardiovascular adaptations that improve exercise capacity, stamina, and promote muscle strength [3,4]. The minimum amount of time adults should spend on moderate to high-intensity physical activities is about 30 min/day [5]. Despite these facts, most people find it difficult to devote time to regular exercise due to typically long workdays that consume 8 to 9 h/d leaving little time for regular leisure-time exercises [6].

The growing involvement of technology and automation in the work environment increases the tendency toward less physically active occupational practices [7,8]. Consequently, increased body mass, decreased physical and work efficiency, increased risk of health problems, increased rates of occupational injuries, and increased mortality have been observed in adults [9,10,11,12,13,14,15,16].

Previous researches on active workspaces have reported positive outcomes over a short period, although the results occasionally depended on the type of work performed. Some studies have shown that walking while working leads to an increase in energy expenditures for many individuals [17,18]; however, while mouse use and typing tasks resulted in more errors over a more extended period of time [19,20,21,22], these activities did not affect mental performance [19,20,23].

Cycling and stepping while working may lead to increased energy expenditures when compared to sitting or walking [19,24]. Nevertheless, the use of more intensive cycling leads to greater numbers of task performance errors [21]. The decline in work performance has been suggested to arise from upper body motions which interfere with the arm stability required for delicate motor tasks [21]. From current research on the impact of physical exercise on psychological and mental processes, moderate steady-state aerobic activities for one hour improved mental performance through a simplified processing of information [25]. There are also long-term benefits of increasing physical activity while at work and these include many psychosocial and physical effects [26]. These can indirectly impact work productivity and directly create a steady and dedicated labor force, provide more attractive jobs when recruiting a new workforce, and improve public relations.

The psychosocial influences related to applying active workplaces include positive relationships between increasing physical activity, the quality of work, and overall work performance [27]. Some researchers concluded that work stress decreased when walking on a treadmill compared to performing work in a seated position [18]. Therefore, a direct effect on work efficiency through innovative workplace design is multifactorial. Increasing physical activity is also related to decreased leave time due to illness which indirectly improves job productivity.

Since there has been overwhelming evidence linking health benefits and physical activity, the goal for companies and human factor experts is to provide practical solutions to increase physical activity at work. However, reducing physical inactivity at work has been challenging due to the increasing number of computer jobs that require sitting for many hours [28]. There have also been increased concerns regarding the possible negative impacts on output when using active workplaces [18], although studies have confirmed that there is no negative impact on performance when walking while typing [29].

Electroencephalography (EEG) is a method of recording the electrical activity of the brain. Several studies showed that EEG could predict low performance owing to changes in mental work [30,31]. Other studies examined the differences in EEG waves during the implementation of continuous and demanding tasks, in which the most memorable event was to increase the theta strength of the EEG on the frontal cortex. This increase has been reported in tasks involving visual search [32], viewing 3D display [33], and loading of working memory. Furthermore, the decrease in the alpha power of EEG has been reported during difficult and cognitive tasks. This decrease occurred in the frontocentral and parietal regions [34,35]. Zhao et al. [36] showed a significant increase in the alpha and theta powers of EEG and a significant reduction in the beta power in different areas of the scalp. They also reported that the beta power decreased in the frontal regions. Moreover, Jap et al. [37] “showed that the stable theta activities led to a decrease of alpha activity in tasks of driving. However, other researchers have also found an increase in alpha activity in train drivers who were sleepy enough to fall asleep while driving [38,39]. Furthermore, increased beta activity has also been related to the alertness level and decreases during drowsiness [40]. Lal and Craig [41] also found an increase in alpha and beta activities during fatigue”.

Physical activity also positively affects the flexibility of the brain through facilitating neurogenerative, neuroprotective, and neuroadaptive processes, and promotes cognition [42], since the combination of mental and physical activity catalyzes cognitive development [43]. Therefore, introducing and/or promoting health interventions in the work environment can ultimately be beneficial [44]. One such intervention was a pedaling exercise, which was found to be practical and contributed to burning extra calories during the day [45].

Therefore, the objective of this study was to investigate the effects of physical activity on human performance at work. In this study, a specific assembly task was assessed while cycling at a stationary workstation. Task performance measures were based on completion time and error percentage, workload perception (subjective workload rating, subjective body discomfort rating), and physiological responses (brain electroencephalographic (EEG) responses and heart rate variability (HRV)) and were geared toward determining whether physical activity, in the form of cycling, affected task performance, workload perception, and physiological responses. The results from this study could help assess the utility of compact under-the-desk stationary bikes in seated workstations in an effort to promote a healthy work environment.

## 2. Materials and Methods

### 2.1. Study Design

The study used “the experimental study design for achieving the determined objectives, integrating into a quantitative approach. The rationale for selecting this particular study design is twofold, such as it is found to provide results in an easy to comprehend form and that too statistically [46]. Secondly, this research design has been effective in deriving concrete and complete results for the other similar researches [47]”. “Effect size was computed by eta-squared (η^2^) as shown in Table 1, Table 2, Table 3 and Table 4, and deemed as: without effect if 0 < η^2^ ≤ 0.04; minimum if 0.04 < η^2^ ≤ 0.25; moderate if 0.25 < η^2^ ≤ 0.64 and; strong if η^2^ > 0.64. To compute the sample power, it was assumed as inputs an expected medium/moderate effect size (for instance, f = 0.25), 5% of error probability for 95% of power, two groups (i.e., assembly task with cycling and the other assembly task without cycling)”, correlation among repeated measures of 0.5 and nonsphericity correction of 1. These inputs yielded a sample size of at least 15 participants in each level, which is less the recruited participants in the experiment.

### 2.2. Participants

For this study, 16 healthy males volunteered to participate: mean age, 30.2 ± 2.6 years; mean weight, 74.43 ± 14.87 kg; mean stature height 168.84 ± 4.72 cm; and body mass index, 26.06 ± 4.62 kg/m^2^. The participants were all graduate students and were experienced in using computers. None of them had self-reported musculoskeletal complaints or problems. All participants were instructed to have a full night’s sleep and to avoid cigarettes and caffeine in the 6 h preceding the tests. Before testing, all participants provided written consent and the protocol and procedures were explained verbally to all students.

### 2.3. Equipment

**Figure 1:**
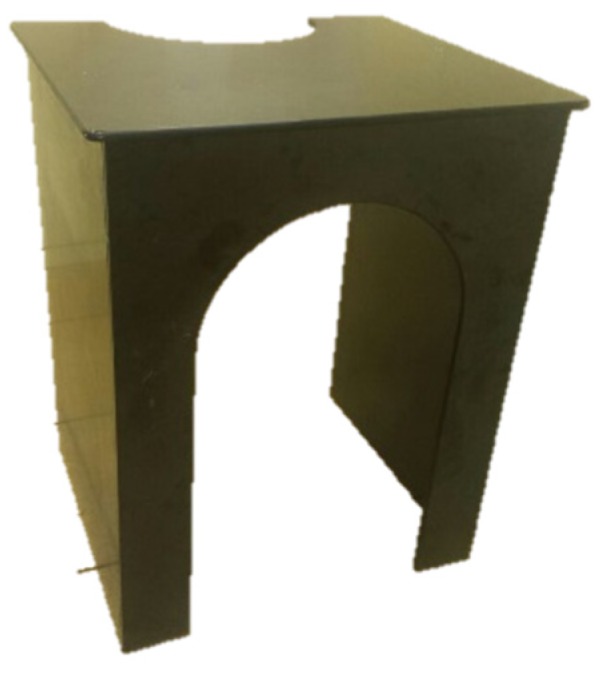
Wooden desktop workstation.

Two identical traditional Microsoft computer keyboards that contained 103 keys and 13 screws were used for the assembly task (one for the assembly and the other one as a reference). A stopwatch was used to record the assembly task time. A commercially available upright exercise bicycle (ergometer) was used for cycling. A wooden desktop (depth = 0.92 m, width = 0.82 m, and height = 1.13 m) specifically designed for the experiment was used as the work station (Figure 1). An eight-channel Biomonitor ME6000, MT-ECG-1 preamplifier, four-channel EEG amplifier for the ME6000, and Mega Win 3.0.1 software (Mega Electronics Ltd., Kuopio, Finland) were used to record physiological signals (four channels to record EEG signals and two channels to record electrocardiographic (ECG) signals). An EMOTIV EEG headset was used for holding the EEG electrodes in place. Kubios HRV Software v2.2 (University of Western Finland, Kuopio, Finland) was used to compute the HRV. Other materials and equipment included 70% isopropyl alcohol swabs, cotton squares, adhesive bandages, Ag/AgCl disk electrodes for EEG signal acquisition, Ag/AgCl solid adhesive pre-gelled electrodes for ECG signal acquisition (Ambu A/S, Denmark), and a high-viscosity electrolyte gel to act as an active electrode.

### 2.4. Assembling Task

**Figure 2:**
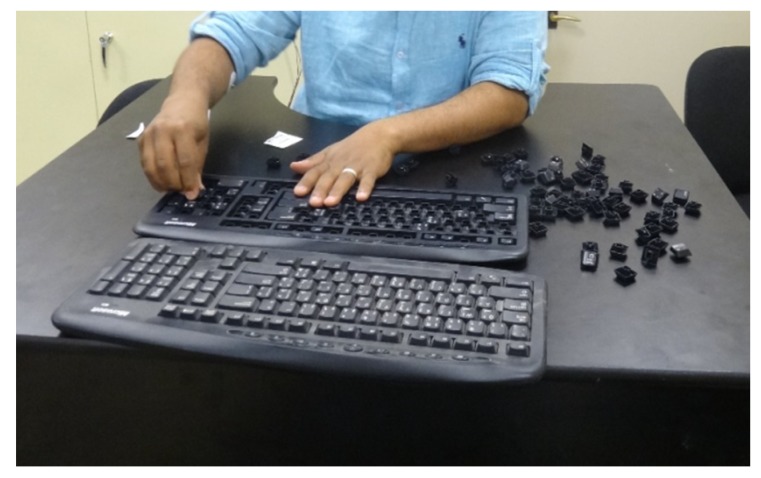
Assembling task.

Each participant was asked to assemble the traditional Microsoft keyboard during each experimental session. An assembled traditional keyboard was provided at each experimental session as a reference guide for participants. Both keyboards were placed on the desktop workstation, as shown in Figure 2, and participants were instructed to assemble the keyboard at their own pace. Additionally, participants were told not to correct any errors that might have occurred during the assembly process. To quantify assembly performance, both assembly time and the number of errors were calculated. This particular task was selected because it includes demanding cognitive operations such as decision-making, visual searching, comparison, memory, concentration, judgment, and placing keys in their correct locations.

### 2.5. Experimental Setup and Procedures

**Figure 3:**
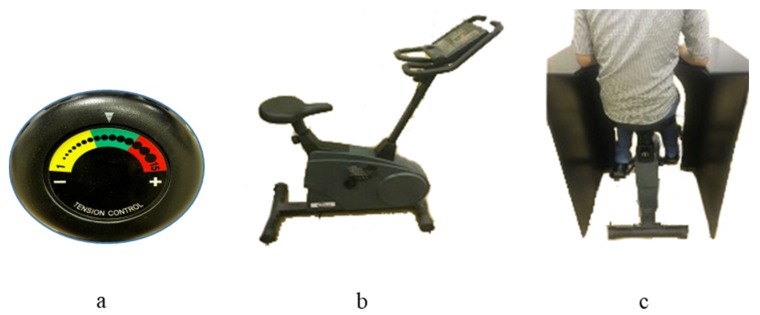
Experimental setup: (**a**) tension control; (**b**) stationary bike; (**c**) adjusting work station.

The laboratory environment had an average temperature of 23.8 °C, a relative humidity of 30.6%, and constant lighting. The innovative experimental site was guaranteed to have no vibrations or strong odors while the tasks were taking place. The experimental setup configuration, along with the ergometer, is shown in Figure 3. The cycling device resistance has a tension control having levels range from yellow to green to red, with yellow being the lowest resistance intensity and red being the highest. The device was adjusted in the center of the green region. Participants pedaled at their preferred speed 15 km/h, or at 25 km/h. The seat height was adjusted by the participants so that their elbows were on the desk surface; however, the desk height and depth remained fixed.

The following protocol was used to perform the tasks. Under experimental conditions, the participants were instructed to assemble a keyboard four times: pedaling at their preferred speed, at 15 km/h, at 25 km/h, or without pedaling. The participants were introduced to the simulated office workstation and the pedal exercise was described to them. Each participant filled out a demographic questionnaire. Afterward, anthropometric measures were taken and the participants were told to adjust the seat to their preference until they felt comfortable. They were then instructed on how to use the bicycle. Each participant then randomly began the four experiments, each one on a different day. After placing the electrodes on the participants, EEG and ECG signals were measured under rest conditions for 5 min (pre). While the participants cycled, a stopwatch was used to record the time from the start of keyboard assembly until completion. After finishing the experiment, the signals were measured again for 5 min (post), and the participants were instructed to fill out the NASA Task Load Index (TLX) rating scale and body discomfort questionnaire.

### 2.6. Response Measures

#### 2.6.1. Electroencephalography (EEG) Signal Responses

The electrode positions were determined according to the 10–20 international standards system for EEG electrode placement [48] (Andreassi, 2010). EEG signals were recorded from the right side of the head at the frontal cortex (positions F3 and F4) which are responsible for attention, judgment, and motor planning. The electrodes were held in place using the EMOTIV headset. The EEG data were amplified and recorded using the Mega Win 3.1b-13 system (Mega Electronics Ltd., Kuopio, Finland) at a sampling rate of 1000 Hz. The recorded EEG signals were visually monitored at all times for any suspicious artifacts. Matlab 2015b and Statistical Package for Social Sciences (SPSS) Version 22 (IBM, Armonk, NY, USA) were used to preprocess and analyze the raw data.

The artifact subspace reconstruction method was used to remove nonstationary high-variance signals from the recorded raw EEG signals and to rebuild missing data using a spatial mixing matrix, assuming volume conduction [49]. This method used a 5 min recording of EEG signals during eye blinking, chewing, and other facial movements, along with 10 s of recorded EEG signals from each trial while a participant lifted a box. Next, a low-pass four-pole elliptic filter with a cutoff frequency of 50 Hz was used to remove the power line noise and any other high-frequency noises. The filtered EEG signals were then separated into their bands (θ, α, and β) using a multilevel discrete wavelet transform. The multilevel discrete wavelet transform (DWT) was utilized to extract the EEG waves, where the DWT included the sub-band of the signal. A DWT that uses Debauches 4 with four levels were employed. Finally, the frequency included in each DWT level was identified by a fast Fourier transform.

The EEG frequency bands of interest are the θ band at 4–8 Hz, the α band at 8–13 Hz, and the β band at 13–30 Hz. Theta and lower α activities may be related to attention, cognition, and memory [50]. Theta is also associated with workload and other cognitive processes such as self-monitoring [51]. The α and β bands are all associated with movement and sensorimotor areas [52,53,54]. The response variables associated with the EEGs and those selected as mental workload indicators were EEG powers of θ, α, β, θ/α, β/α, and (α + θ)/β indices.

#### 2.6.2. Electrocardiographic (ECG) Response Analysis

Standard procedures were followed for the placement of the 3 ECG Ag/AgCl solid adhesive pre-gelled electrodes on the participants’ chests [55]. “The ECG signals were recorded using Mega Win software at a sampling rate of 1000 Hz. These data were obtained using the MT-ECG-1 preamplifier (Mega Electronics Ltd., Kuopio, Finland) connected to two-channel of Biomonitor ME6000. The ECG signal was imported and read using the code in MATLAB TM. In the next step, the data was extracted into a text file from the MATLAB for estimating HRV indices. Then, Kubios HRV Software v2.2 was used for processing the ECG signals” [56]. The recorded ECG signals were pre-processed to eliminate artifacts by removing interbeat (R-R) intervals that differed by more than 25% between two consecutive R-R intervals. The removed R-R intervals were replaced via conventional spline interpolation, meaning the length of the data remained unchanged (i.e., the same number of beats). The smoothness prior method with a lambda value of 1000 was used for the removal of disturbing low-frequency baseline trend components [57,58].

The time-domain parameters of the HRV calculated in this study were the mean R-R intervals (mRR), the standard deviation of all R-R intervals (SDRR), the standard deviation of heart rate (SDHR), root mean square of successive differences (RMSSD), NN50 (number of pairs of adjacent normal-to-normal (NN) intervals differing by more than 50 ms), and pNN50 (NN50 count/total NN count). The frequency-domain parameters of the HRV calculated in this study were the very low frequency (VLF), low frequency (LF), high frequency (HF), and LF/HF. Absolute power was calculated as milliseconds squared divided by cycles per second (ms^2^/Hz).

#### 2.6.3. Performance Measures

For each participant, the completion time of the assembly task and the number of errors in the final assembly were measured to assess performance. One error was any wrong insertion of a keyboard button. The error in this study is defined as either inserting the buttons in an incorrect direction or incorrect place. The percentage of errors was calculated as the number of errors divided by all keys of the keyboard.

#### 2.6.4. Subjective Workload Ratings

The NASA-TLX [59] was used to subjectively assess the perceived workload and involved six subscales: “physical demands, mental demands, performances, temporal demands, effort, and frustration levels”.

#### 2.6.5. Subjective Body Discomfort Ratings

In this study, a discomfort survey questionnaire by the Vyas study [60] was used. Participants were asked to rate the feeling of discomfort for twelve body parts (neck, shoulders, elbows, upper back, lower back, forearms, wrist/hands, hips, thighs, knees, legs, ankles/feet) on a scale from 0 to 10 (where 0 represented no discomfort, and 10 represented severe discomfort).

### 2.7. Experimental Design

A counterbalance repeated measures analysis of variance (ANOVA) design with two independent variables and six response variables (EEG indices, heart rate variability, task completion time, error percentage, subjective workload ratings (NASA-TLX), and total body discomfort scores) was used in this study. The independent variables were the session test (pre vs. post) and the assembling methods (without cycling, cycling at 15 km/h, cycling at the participant’s preferred speed, and cycling at 25 km/h). A counterbalanced one-way repeated measures ANOVA design with one independent variable (assembling methods) was used to study the four response variables (task completion time, error percentage, subjective workload ratings (NASA-TLX), and total body discomfort scores) since it was performed after the experiments. A two-way repeated measures ANOVA design, with two independent variables (session time and assembling methods) and two dependent variables (EEG indices, heart rate variabilities) was also used.

### 2.8. Data Analysis

Statistical analysis of the data was performed with SPSS software version 22 (SPSS Inc., Chicago, IL, USA). In all analyses, *p*-values < 0.05 were considered statistically significant.

## 3. Results

### 3.1. EEG Signals

The EEG power was measured at frontal regions F3 and F4 and analyzed using two-way ANOVA tests. The EEG indices were divided into two groups: the basic (relative power) and ratio indices. The basic indices can contradict one another; therefore, the ratio indices were calculated to strengthen the differences. The basic indices that were selected for analyzing the power of the EEG were (θ, α, and β). The power ranged from 4 to 30 Hz, as described in Section 2.6.1. The ratio indices θ/α, β/α, and (α + θ)/β were also analyzed. The 1 min EEG data segments at the pre- and post-sessions were chosen for the analysis. The repeated design results of the EEG data are listed in Table 3 which reveals that the overall significance is from the session times. It shows that there is a significant difference for session time (pre and post) in alpha and beta EEG signals. The mean (standard deviation) and the statistical values (F and *p*-values) of the EEG indices for the two-session tests and four assembly methods are summarized in Table 3. There were no significant effects of the assembly methods on the relative EEG power for the θ, α, and β, as well as the θ/α, β/α, and (α + θ)/β logarithm. The results indicated that only the session test has a significant effect on the alpha and beta EEG power.

### 3.2. Electrocardiographic (ECG) Response Analysis

**Figure 4:**
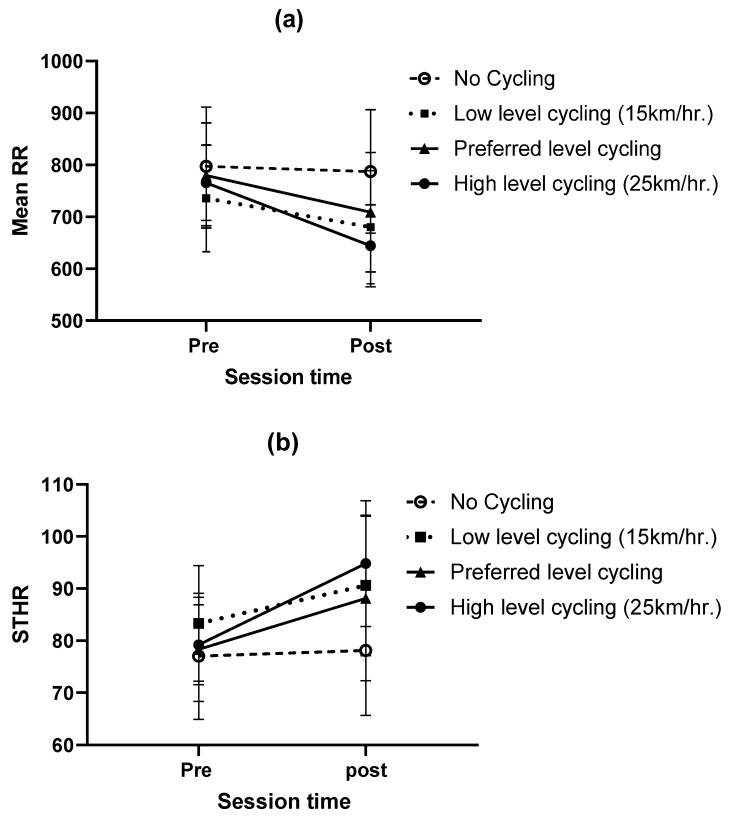
Interaction effects between the session and the assembly methods on: (**a**) mean interbeat (R-R) and (**b**) standard deviation of heart rate (SDHR).

The repeated design test results of the HRV data at two sessions (pre and post) for four conditions are listed in Table 4. The time-domain parameters of the HRV (R-R, SDRR, RMSSD, NN50, and PNN50) were used to evaluate the differences between the assembly methods. The interaction between the session time (pre and post) and assembly methods had a significant effect on the mRR interval, and STD_HR: F = 15.64, *p* < 0.001; and F = 17.791, *p* < 0.001, respectively, as listed in Table 4. The SDHR increased, and the mRR decreased after the participants executed the assembly task while performing the pedaling exercises. In contrast, they remained approximately the same for the participants when executing the assembly without performing the pedaling exercise as shown in Figure 4.

In addition, there was a significant difference in the mRR, and the STD_HR between the session time (pre and post) regardless of the assembling methods: F = 55.7, *p* < 0.001; F = 43.68, *p* < 0.001, respectively.

### 3.3. Completion Time, Body Discomfort Rating, and Error Analysis

**Figure 5:**
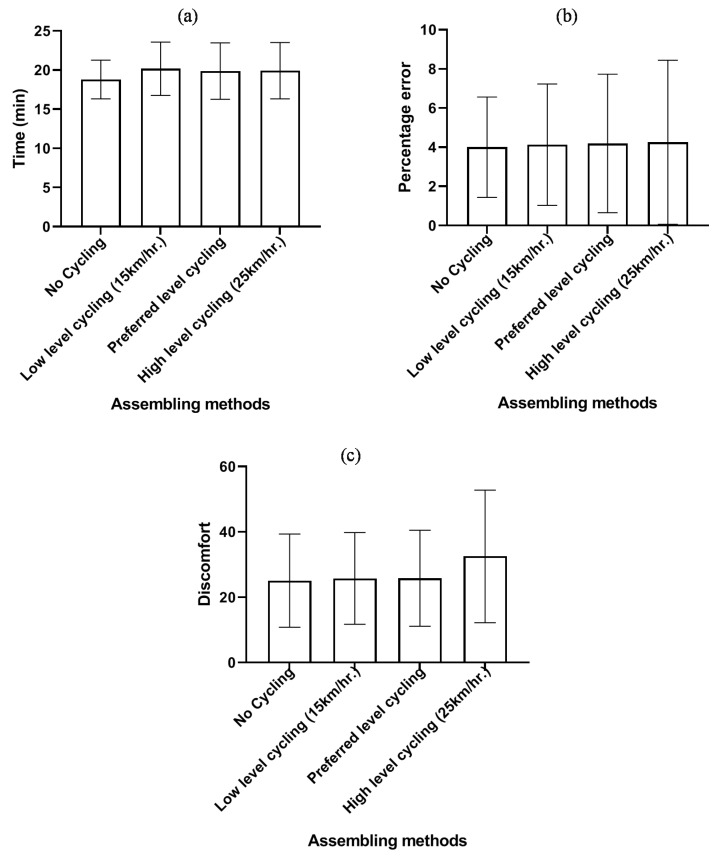
Mean (**a**) time, (**b**) percentage error, and (**c**) discomfort during cycling conditions.

Table 1 shows that there was no significant difference in the completion time, the body discomfort rating or the number of errors. Figure 5 shows the means of error and time in the cycling conditions.

### 3.4. Subjective Workload Analysis

The NASA-TLX questionnaire was used to measure the subjective workload. The NASA-TLX contains six subscales: “mental demand, physical demand, temporal demand, performance, effort, and frustration level”. The average score of the NASA-TLX was calculated using the NASA-TLX Online Tool (Version 0.06, NASA, Washington, DC, USA) provided by Sharek [61]. The repeated measure test results of the NASA-TLX data at two sessions (pre and post) for all cycling conditions are listed in Table 2.

Table 2 shows that there was a significant difference in the scores of the NASA-TLX, the physical demand, and effort between the assembling methods: F = 5.91, *p* < 0.009; F = 14.507, *p* < 0.001; and F = 8.678, *p* < 0.002, respectively.

## 4. Discussion

One of the challenges that manufacturing companies are dealing with currently is the ability to avoid quality errors in manual assembling tasks and operations, especially since many tasks and activities are done at the same time. The job proposed in the study includes demanding cognitive processes that are described in an assembly task. For such a task, any other physical effort added could affect the partaker’s performances. ECG and EEG are relevant response variables in these tasks. University students executed a keyboard assembly task, and the study investigated the partaker’s performance in executing the assembly tasks and whether or not it would be affected if they performed pedaling exercises simultaneously. In the experiment, different measures were studied when introducing the bicycle pedaling practice to the workload and performance while achieving the assembly task.

This study evaluated whether the performance of the participants was affected by pedaling and pedaling speed while performing an assembly task. In this study, 16 students from King Saud University completed the assembly of a keyboard using 4 assembly methods, each performed on different days. Various measurements were used to investigate the effect of the bicycle pedaling exercise on human workload and performance during the assembly experiment. The results determined that: (1) cycling had a significant effect on alpha and beta EEG signals in each session time (pre and post); (2) for ECG signals the interaction between the session time (pre and post) and cycling condition had a significant effect on the mRR interval. In addition, there was a significant difference in the mRR interval between the session time (pre and post) regardless of the assembly methods; (3) cycling did not have a significant impact on completion time; and (4) cycling did not have a significant effect on body discomfort rating or the number of errors.

The most predictive and reliable index to estimate mental fatigue is the EEG signal. A change in brain arousal involves specific changes in oscillatory brain activity and an EEG signal can reflect the fluctuations of the alertness levels. Many studies have reported an increase in the θ frequency band over the frontal cortex. In addition, the α band has been reported to decrease during complex and cognitively demanding tasks. The increase in the θ power has been reported in studies involving visual searching [32] and working memory load [62,63,64,65] with decreases in the θ band in different scalp areas, such as the parietal and the front-central regions [34,35]. Other researchers Zhao et al. (2012) [36] showed that the relative power of the α and θ rhythms significantly increased, whereas the relative power of the β rhythm significantly decreased in different scalp regions. In addition, the β rhythm was significantly decreased in the frontal regions. In this study, there was a significant difference for session times (pre and post) in α and β powers. In addition, there was no significant effect for the two-way interaction between session time and cycling condition, or for cycling condition on the power of EEG for the θ, α, and β rhythms and the θ/α, β/α, and (α + θ)/β logarithms. However, there were no significant differences in most conditions. The results of the EEG response indicated that the process of reaching the mental workload was not displayed. This finding is possible because the task time was insufficient for the participants to reach a certain fatigue level, or those participants had sufficient typing experience. Additionally, no comparable previous studies exist in which EEG measurements were obtained while performing the pedaling exercises.

Based on the ECG signals, the interaction between the session time (pre and post) and cycling condition had a significant effect on the mRR interval, and the SDHR. The mRR interval decreased by 7.57%, 9.08%, and 15.86% after the participants performed the assembly at 15 km/h, their preferred speed, and at 25 km/h respectively, and the mRR interval remained approximately constant without pedaling. These decreases occurred because of the physical effort of pedaling. The results of this study were consistent with a previous study by Elmer and Martin (2014) [66], and other researchers Straker et al. (2009) reported that heart rate values were 25% greater when pedaling and typing than when sitting and typing [21]. (Straker et al., 2009) They also evaluated the effects of a cycling workstation (a computer desk and an upright exercise cycle) on the physiological responses and computer operation of office workers [21]. There were no significant changes in the SDRR, RMSSD, NN50, pNN50, and frequency domain measurements for any cycling conditions.

Cycling conditions did not affect body discomfort ratings and some errors. However, some commented that it was difficult to assemble the keyboard due to the simultaneous physical movement in the lower and upper limbs. This conclusion agrees with previous studies where pedaling resulted in a decline in performance related to tasks using a mouse [21,67,68].

Based on the NASA-TLX score, a difference between assembly methods was observed. Therefore, the average physical workload NASA-TLX scores increased by 42.5% when assembling the keyboard while pedaling. Performing the assembly while pedaling only increased the physical workload. This may be due to the type of exercise. Previous studies have reported mixed results for computer operations (typing time, speed, word count, and/or accuracy) with cycling workstations [21], treadmill workstations [21,22,69,70], and other novel workstation modalities [71] when compared to sitting. The results of this study were in agreement with a previous study which showed that a cycling work station might offer a viable option for increasing the physical activity of a desk-bound worker without compromising typing performance [66].

In physiology, “the autonomic nervous system, including the sympathetic and parasympathetic nervous systems control the cardiovascular system. Human heart rates violently fluctuate during mental stress. Heart rate is primarily controlled by the autonomic nervous system and it is increased by shifting the sympathovagal balance during mental fatigue”. In general, the sympathetic nervous system is activated during intense states. The parasympathetic nervous system is activated in states of mental peace; therefore, the physiological response be opposite to the sympathetic nervous system. This study showed no significant changes in the frequency domain measurements for cycling conditions. Thus, the predominant activity of the autonomic nervous system of the participants did not change to sympathetic activity from parasympathetic activity after the task.

## 5. Conclusions

In conclusion, there were no detrimental effects from pedaling while performing the assembly tasks. Therefore, performing moderate physical activity is beneficial to a person’s health, safety, and well-being.

### Limitations

Despite the authors’ repeated attempts to recruit females by distributing flyers and pamphlets in the girls’ section, only male participants were recruited for the study. Another restriction on the finding pertains to the participants’ pool recruited for this laboratory study. The participants have recruited from the college student population, and none of the participants had significant prior office work experience. However, the results presented herein may apply to the experienced community for similar office tasks.

## Figures and Tables

**Table 1:** Mean (standard deviation) for time, discomfort, and error.

Indices	Assembling Methods	Mean (SD)	b	
			η^2^	*p*-Value
Time	No Cycling	18.8 (2.5)	0.387	0.09
	Low level cycling (15 km/h)	20.2 (3.4)		
	Preferred level cycling	19.9 (3.6)		
	High level cycling (25 km/h)	19.9 (3.6)		
Error	No Cycling	4.0 (2.6)	0.005	0.99
	Low level cycling (15 km/h)	4.1 (3.1)		
	Preferred level cycling	4.2 (3.5)		
	High level cycling (25 km/h)	4.3 (4.2)		
Discomfort	No Cycling	25.1 (14.3)	0.199	0.39
	Low level cycling (15 km/h)	25.8 (14.1)		
	Preferred level cycling	25.8 (14.7)		
	High level cycling (25 km/h)	32.5 (20.3)		

b: assembly methods, η^2^: partial eta squared.

**Table 2:** Subjective workload (NASA-TLX).

Indices	Assembling Methods	Mean (SD)	b	
			η^2^	*p*-Value
NASA_Average	No Cycling	35.1 (12.7)	0.577	**0.009**
	Low level cycling (15 km/h)	40.1 (14.1)		
	Preferred level cycling	46.4 (11.8)		
	High level cycling (25 km/h)	48.9 (12.2)		
Scale_Mental	No Cycling	57.6 (24.5)	0.141	0.56
	Low level cycling (15 km/h)	53.7 (24.5)		
	Preferred level cycling	63.5 (22.8)		
	High level cycling (25 km/h)	59.1 (22.1)		
Scale_Physical	No Cycling	28.4 (16.7)	0.747	**0.00**
	Low level cycling (15 km/h)	46.7 (22.6)		
	Preferred level cycling	54.3 (21.1)		
	High level cycling (25 km/h)	65.1 (18.0)		
Scale_Temporal	No Cycling	48.7 (14.9)	0.170	0.47
	Low level cycling (15 km/h)	49.8 (13.4)		
	Preferred level cycling	50.4 (15.2)		
	High level cycling (25 km/h)	53.3 (13.8)		
Scale_Performance	No Cycling	24.8 (19.1)	0.086	0.25
	Low level cycling (15 km/h)	27.4 (19.0)		
	Preferred level cycling	32.6 (14.4)		
	High level cycling (25 km/h)	24.1 (11.8)		
Scale_effort	No Cycling	33.8 (22.0)	0.466	**0.002**
	Low level cycling (15 km/h)	42.9 (24.5)		
	Preferred level cycling	46.6 (24.6)		
	High level cycling (25 km/h)	64.6 (19.8)		
Scale_Frustration	No Cycling	20.4 (21.9)	0.008	0.72
	Low level cycling (15 km/h)	24.1 (24.9)		
	Preferred level cycling	26.5 (19.8)		
	High level cycling (25 km/h)	26.9 (19.7)		

SD: Standard deviation, TLX: Task Load Index, bolded *p*-values indicate statistical significance, b: assembly methods, η^2^: partial eta squared.

**Table 3:** Mean (standard deviation) values of power of EEG before and after assembly task with cycling conditions.

	Parameters	Mean (SD)								A*B		A		B	
	Session Time	Pre				Post				η^2^	*p*	η^2^	*p*	η^2^	*p*
	Assembly Methods	No Cycling	15 km/h	Preferred	25 km/h	No Cycling	15 km/h	Preferred	25 km/h						
EEG responses	α	598.8 (524)	568.4 (409.8)	619 (416.7)	628.3 (471)	760.1 (628.2)	891.7 (765.9)	799.9 (399.5)	773.5 (418.2)	0.065	0.824	0.313	**0.02**	0.021	0.964
	θ	300.1 (127.4)	314.5 (223)	406.4 (358)	255 (90.6)	289.7 (114.7)	381.1 (255.5)	258.3 (62.3)	643.4 (103.5)	0.188	0.424	0.081	0.27	0.105	0.683
	β	335.2 (152.9)	300.6 (121.1)	358.7 (133.4)	301.2 (111.1)	450.6 (218.8)	361.2 (113.4)	438.6 (211.9)	482.7 (279.8)	0.166	0.486	0.468	**0.002**	0.274	0.229
	θ/α	0.77 (0.46)	0.8 (0.5)	0.9 (0.8)	0.7 (0.5)	0.7 (0.51)	0.7 (0.6)	0.5 (0.3)	0.9 (1.0)	0.241	0.293	0.115	0.184	0.106	0.68
	β/α	0.87 (0.65)	0.8 (0.5)	0.8 (0.4)	0.8 (0.6)	1.0 (1.1)	0.8 (0.4)	0.8 (0.7)	0.8(0.6)	0.109	0.109	0.005	0.788	0.040	0.908
	(α + θ)/β	2.97 (1.54)	2.8 (1.7)	2.8 (1.2)	2.9 (1.5)	2.74 (1.4)	2.6 (1.6)	2.5 (1.01)	3.2 (1.2)	0.084	0.757	0.030	0.509	0.154	0.521

A: session time, B: assembly methods, η^2^: partial eta squared, *p*: *p*-value, EEG: electroencephalogram, SD: standard deviation, bolded *p*-values indicate statistical significance.

**Table 4:** Mean (standard deviation) values of heart rate variability before and after assembly tasks with cycling conditions.

	Parameters	Mean (SD)								A*B		A		B	
	Session Time	Pre				Post				η^2^	*p*	η^2^	*p*	η^2^	*p*
	Assembly Methods	No Cycling	15 km/h	Preferred	25 km/h	No Cycling	15 km/h	Preferred	25 km/h						
Time Domain Measures	R-R (ms)	797.1 (114.5)	735.8 (103.1)	779.7 (101.3)	765.7 (72.6)	787.5 (118.8)	680 (109)	708.9 (114.9)	644.3 (79)	0.783	**0.00**	0.788	**0.00**	0.439	0.051
	SDRR (ms)	27.5 (12.7)	28.8 (15)	28.5 (14.2)	25.8 (7.4)	29.4 (15.6)	28.6 (12.2)	35.6 (39.8)	23.1 (11.4)	0.161	0.501	0.022	0.573	0.169	0.476
	SDHR	77 (12.1)	83.3 (11.1)	78.3 (10)	79.2 (7.7)	78.1 (12.4)	90.6 (13.5)	88.1 (15.8)	94.8 (12.1)	0.806	**0.00**	0.745	**0.00**	0.405	0.072
	RMSSD (ms)	24.3 (14.3)	23.6 (16.5)	24.9 (17.2)	19.7 (4.5)	25.8 (15.2)	21.9 (12.5)	32 (53)	16.9 (14.6)	0.093	0.723	0.006	0.766	0.248	0.279
	NN50	5.3 (7.8)	6.8 (10.4)	6.5 (9.3)	1.7 (1.7)	5.9 (7.2)	5 (7)	6.3 (9.1)	3.6 (9.6)	0.158	0.509	0.001	0.889	0.153	0.524
	pNN50 (%)	8.2 (12.9)	7.9 (13.9)	9.6 (14.5)	2.2 (2.2)	9 (12.1)	6.6 (10.4)	8.6 (12.2)	4.1 (10.1)	0.161	0.499	0	0.941	0.206	0.376
	VLF (ms^2^)	110.4 (112.5)	110 (144.5)	121.5 (181.7)	101.5 (96.5)	54.8 (50.8)	116.1 (176.7)	124.3 (196.9)	62.6 (73.2)	0.165	0.489	0.069	0.309	0.114	0.653
Frequency DomainMeasures	LF (ms^2^)	378.5 (291.9)	520.4 (570.3)	556.2 (587.4)	378.7 (379.7)	635.9 (620.9)	619.8 (578.9)	686 (512.4)	345.8 (338.2)	0.142	0.56	0.086	0.252	0.130	0.597
	HF (ms^2^)	335.3 (446.1)	295.5 (461.2)	419.2 (773.7)	152.3 (105.7)	399.1 (559.7)	211.5 (263.5)	415.9 (442.6)	95.3 (98.1)	0.282	0.216	0.005	0.788	0.369	0.102
	LF/HF (ms^2^)	3.5 (4.2)	4.5 (4)	3.7 (3.7)	3. 9 (3.7)	3.7 (4.4)	7.8 (10.4)	7.1 (8.7)	6.6 (5.5)	0.127	0.608	0.249	0.041	0.140	0.565

A: session time, B: assembly methods, η^2^: partial eta squared, *p*: *p*-value, SD: standard deviation; R-R: interbeat interval; ms: milliseconds; SDRR: standard deviation of all R-R intervals, SDHR: standard deviation of the mRR intervals, RMSSD: root mean square of successive differences, NN50: number of pairs of adjacent normal-to-normal (NN) intervals differing by more than 50 ms, pNN50: (NN50 count/total NN count). VLF: very low frequency, LF: low frequency, HF: high frequency. Bolded *p*-values indicate statistical significance.
